# MRI features of pediatric atypical teratoid rhabdoid tumors and medulloblastomas of the posterior fossa

**DOI:** 10.1002/cam4.5780

**Published:** 2023-03-14

**Authors:** Hsin‐Wei Wu, Chia‐Hung Wu, Shih‐Chieh Lin, Chih‐Chun Wu, Hsin‐Hung Chen, Yi‐Wei Chen, Yi‐Yen Lee, Feng‐Chi Chang

**Affiliations:** ^1^ Department of Radiology Taipei Veterans General Hospital Taipei Taiwan; ^2^ School of Medicine, National Yang Ming Chiao Tung University Taipei Taiwan; ^3^ Institute of Clinical Medicine, National Yang Ming Chiao Tung University Taipei Taiwan; ^4^ Department of Pathology and Laboratory Medicine Taipei Veterans General Hospital Taipei Taiwan; ^5^ Division of Pediatric Neurosurgery, Department of Neurosurgery, Neurological Institute Taipei Veterans General Hospital Taipei Taiwan; ^6^ Department of Oncology Taipei Veterans General Hospital Taipei Taiwan; ^7^ Department of Medical Imaging and Radiological Technology Yuanpei University of Medical Technology Hsinchu City Taiwan

**Keywords:** atypical teratoid rhabdoid tumor (AT/RT), embryonal brain tumor, magnetic resonance imaging (MRI), medulloblastoma, pediatric brain tumor

## Abstract

**Background:**

Atypical teratoid rhabdoid tumor (AT/RT) occurs at a younger age and is associated with a worse prognosis than medulloblastoma; however, these two tumor entities are mostly indistinguishable on neuroimaging. The aim of our study was to differentiate AT/RT and medulloblastoma based on their clinical and MRI features to enhance treatment planning and outcome prediction.

**Methods:**

From 2005–2021, we retrospectively enrolled 16 patients with histopathologically diagnosed AT/RT and 57 patients with medulloblastoma at our institute. We evaluated their clinical data and MRI findings, including lesion signals, intratumoral morphologies, and peritumoral/distal involvement.

**Results:**

The age of children with AT/RT was younger than that of children with medulloblastoma (2.8 ± 4.9 [0–17] vs. 6.5 ± 4.0 [0–18], *p* < 0.001), and the overall survival rate was lower (21.4% vs. 66.0%, *p* = 0.005). Regarding lesion signals on MRI, AT/RT had a lower ADC_min_ (cutoff value ≤544.7 × 10^−6^ mm^2^/s, *p* < 0.001), a lower ADC ratio (cutoff value ≤0.705, *p* < 0.001), and a higher DWI ratio (cutoff value ≥1.595, *p* < 0.001) than medulloblastoma. Regarding intratumoral morphology, the “tumor central vein sign” was mostly exclusive to medulloblastoma (24/57, 42.1%; AT/RT 1/16, 6.3%; *p* = 0.007). Regarding peritumoral invasion on T2WI, AT/RT was more prone to invasion of the brainstem (*p* < 0.001) and middle cerebellar peduncle (*p* < 0.001) than medulloblastoma.

**Conclusions:**

MRI findings of a lower ADC value, more peritumoral invasion, and absence of the “tumor central vein sign” may be helpful to differentiate AT/RT from medulloblastoma. These distinct MRI findings together with the younger age of AT/RT patients may explain the worse outcomes in AT/RT patients.

## Graphical abstract

AT/RT occurs in younger patients, has more aggressive clinical behavior and is associated with much worse outcomes than medulloblastoma. On MRI, AT/RT (G‐I) can be differentiated from medulloblastoma (A‐F) by a lower ADC value, more peritumoral invasion, and the absence of the “tumor central vein sign”.

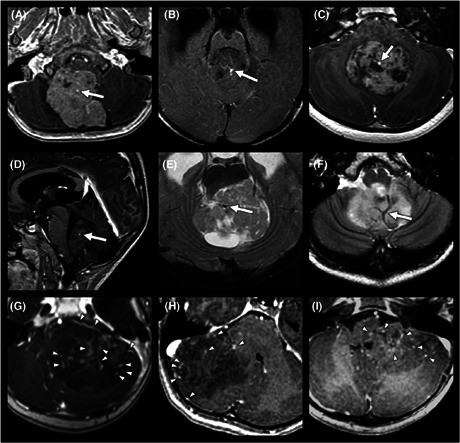

## 1 INTRODUCTION

Embryonal tumors account for approximately 20% of pediatric brain tumors, and they share a common histological feature: dense small round blue cells.^1^, ^2^ According to the 2021 World Health Organization (WHO) CNS5 classification, embryonal tumors can be further classified into “medulloblastoma” and “other CNS embryonal tumors”.^1^ Medulloblastoma is the most common posterior fossa brain tumor in pediatric patients, accounting for approximately 20% of pediatric central nervous system (CNS) tumors and 60% of embryonal tumors.^2^, ^3^ In comparison, atypical teratoid rhabdoid tumor (AT/RT), the leading subtype in the “other CNS embryonal tumor” category, accounts for 1%–2% of pediatric CNS tumors.^1^, ^4^ AT/RT develops at a younger age than medulloblastoma. Approximately 80.5% of AT/RTs occur in children under 3 years of age.^5^ Due to the aggressive behavior of embryonal tumors, AT/RT and medulloblastoma are often treated with trimodality therapy consisting of surgery, chemotherapy and postoperative radiotherapy.^6^ Radiotherapy is generally performed in patients older than 3 years of age due to associated neurocognitive toxicity in infants.^6^, ^7^, ^8^ The prognosis of AT/RT is extremely poor, even though with a slight improvement in recent years. The 4‐year overall survival rate was 43% with AT/RT (the ACNS0333 trial), compared to a 5‐year overall survival rate of 82.3% in those with medulloblastoma (the SJMB03 trial).^9^, ^10^

AT/RT and medulloblastoma have many similar features and are generally indistinguishable on neuroimaging.^5^, ^11^, ^12^, ^13^, ^14^, ^15^ More than half of the AT/RTs are located infratentorially.^14^, ^15^ On unenhanced computed tomography (CT), both tumors appear hyperdense due to the high nuclear‐cytoplasmic ratio.^5^, ^14^, ^15^ On magnetic resonance imaging (MRI), they appear iso‐to‐hypointense compared to the gray matter on T1‐weighted imaging (T1WI), heterogeneous with variable signal intensity on T2‐weighted imaging (T2WI) and show enhancement after contrast administration.^5^, ^16^ Intralesional hemorrhage may occur in both tumor types, although the incidence is higher for AT/RT.^5^, ^14^, ^15^ Diffusion‐weighted imaging (DWI) has been widely used for the differentiation of brain tumors^12^, ^17^; however, both AT/RT and medulloblastoma appear hyperintense on DWI and are reported to have an overlapping apparent coefficient (ADC) value.^12^, ^14^, ^18^ Pretreatment diagnosis is important in treatment planning due to the significantly worse prognosis of AT/RT than medulloblastoma, especially in very young patients, for whom radiotherapy is less suitable. The aim of this retrospective study was to define clinical and MRI features that may help to differentiate AT/RT and medulloblastoma in pediatric patients to allow appropriate treatment planning and improve patient outcomes.

## 2 METHODS

This retrospective study was approved and deemed exempt from individual patient consent for this research project by the institutional review board of our institute. Informed consent to perform imaging examinations, surgery and adjuvant cancer treatment was obtained from each patient or their family.

### 2.1 Patients

From 2005 to 2021, there were 22 pediatric patients with histopathological diagnoses of AT/RT and 70 pediatric patients with medulloblastoma of posterior fossa in our institute. Six AT/RT patients and 13 medulloblastoma patients without presurgical MRI information in our hospital were excluded. In total, 73 pediatric patients (16 AT/RT and 57 medulloblastoma patients) were retrospectively enrolled in this study. We thoroughly reviewed their clinical characteristics, surgical records (gross total removal, nearly total removal, subtotal removal, or partial removal), adjuvant cancer treatments (chemotherapy and radiotherapy), tumor recurrence/progression, and survival.

### 2.2 Presurgical MRI

Conventional MRI was performed with a 1.5T clinical MR scanner (Siemens Medical Solutions; GE Medical Systems; or Philips Medical Systems); the protocol included axial noncontrast T1/T2‐weighted imaging (T1WI/T2WI), axial and sagittal contrast‐enhanced fat‐suppressed T1WI, DWI with *b* values of 0 and 1000 s/mm^2^ applied in three orthogonal directions, and apparent diffusion coefficient (ADC) maps generated automatically by the MRI scanners. T2* gradient‐echo (GRE) or susceptibility‐weighted imaging (SWI for the Siemens instrument, SWAN for the GE instrument) was additionally performed in 15 patients. HWW and FCC reviewed all the images separately. They were blind to the clinical information and pathological diagnosis during the imaging analysis.

**FIGURE 1:**
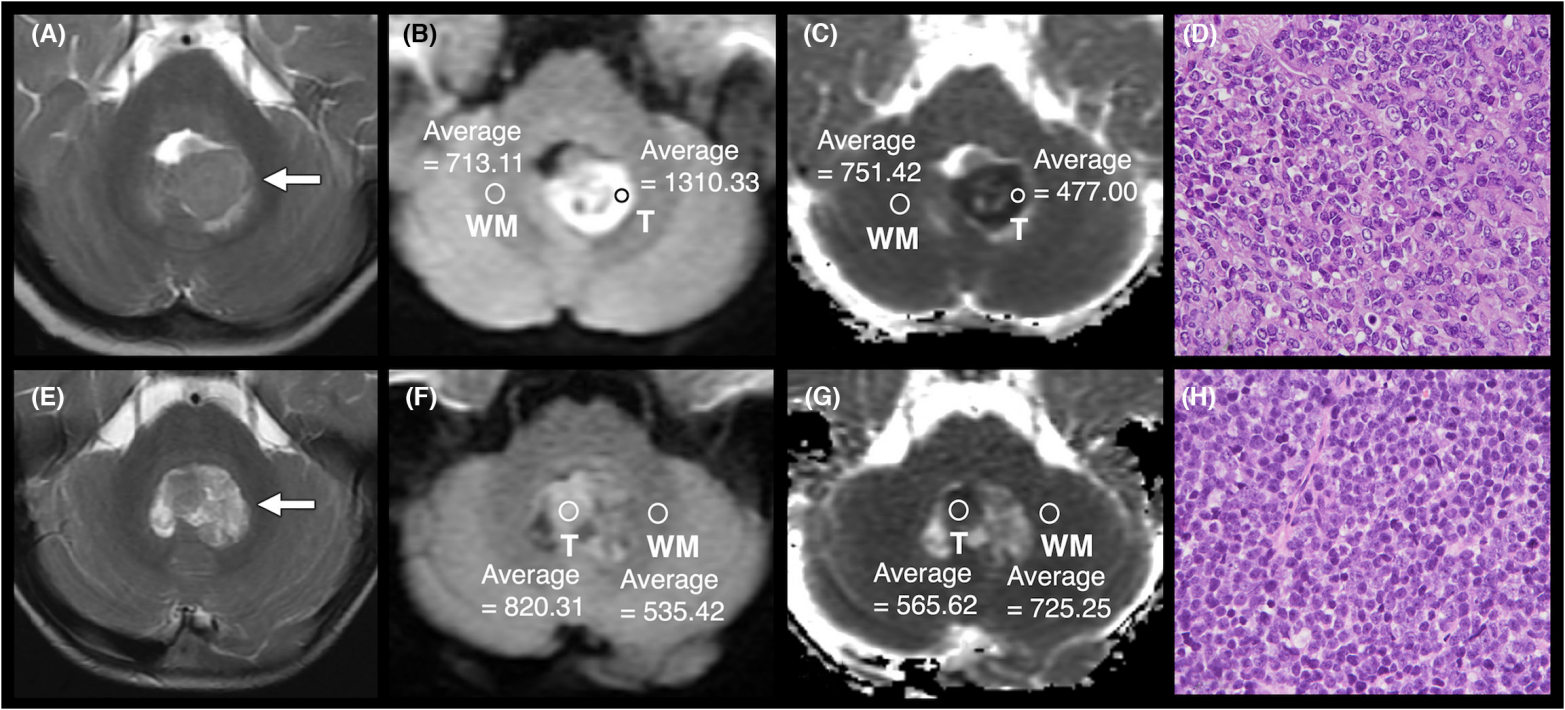
Measurement of DWI and ADC ratios and their corresponding histopathological findings. (A–D) represents a 1‐year‐old girl with AT/RT, and (E–H) represents a 3‐year‐old boy with medulloblastoma. These two patients had similar brain tumor imaging features on T2WI (A, E, arrows). The first tumor ROI (T) was positioned on the homologous area with the highest signal within the solid components on DWI (B, F) and on the area with the lowest signal for ADC calculation (C, G). The second and the third tumor ROIs were obtained on different image sections (not shown). The three tumor ROIs on DWI and the three tumor ROIs on ADC were averaged, respectively. Additional ROIs were placed on homologous areas of normal‐appearing contralateral white matter (WM) on DWI (*b* = 1000 s/mm^2^) (B, F) and for ADC calculation (C, G) to offset the subtle signal differences among the different MR scanners. The AT/RT patient had a DWI ratio of 1.85, an ADC_min_ of 471 × 10^−6^ mm^2^/s, and an ADC ratio of 0.63. The medulloblastoma patient had a DWI ratio of 1.59, an ADC_min_ of 536 × 10^−6^ mm^2^/s, and an ADC ratio of 0.74. (D) The histopathology of AT/RT was composed of tumor cell with vesicular nuclei and most of the tumor cells had prominent nucleolus. Part of the tumor cells had eosinophilic intracytoplasmic inclusion. Nuclear molding was easily identified within the tumor. (H&E staining) (H) The histopathology of medulloblastoma was composed of tumor cells with small blue round cell morphology. (H&E staining).

We analyzed the posterior fossa tumors according to their lesion size and signal intensity of the solid portions on T1WI, T2WI, and contrast‐enhanced T1WI. Absolute ADC values (ADC_min_) were measured by manually positioning regions of interest (ROIs) 10–50 mm^2^ in size using hospital picture archiving and communication system (PACS) workstations. Tumor ROIs were positioned on the homologous area with the lowest signals within the solid components while avoiding areas with necrosis, peritumoral edema, calcification and hemorrhage.^12^, ^13^, ^17^ If the tumor appears in more than three images, three ROIs were placed on different sections then averaged. If the tumor appears in less than three images, a total of three ROIs were placed within the tumor in the avoidance of overlapping.^13^, ^14^ To offset the subtle signal settings of different MRI scanners, an additional region of interest (ROI) was positioned on the homologous area of normal‐appearing contralateral white matter of the cerebellum.^12^, ^13^, ^17^ The ADC ratio was calculated as the solid tumor to contralateral white matter ratio. The DWI ratios (*b* = 1000 s/mm^2^) were obtained in a similar manner but by positioning the tumor ROIs at areas with the highest signals (Figure 1).^12^, ^17^

**FIGURE 2:**
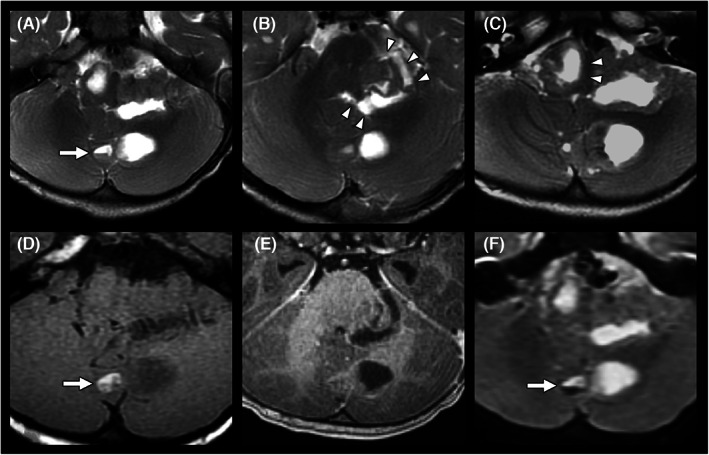
A case of atypical teratoid rhabdoid tumor (AT/RT). A 1‐year boy presented with unsteady gait for 2 weeks. On presurgical MRI, T2WI revealed a multiloculated mass lesion with central necrosis in the posterior fossa (A–C). The lesion showed local invasion into the middle cerebellar peduncle (B, arrowheads) and brain stem (C, arrowheads). The soft tissue components appeared isointense on T1WI (D) and showed good enhancement on contrast‐enhanced T1WI (E). A fluid–fluid level indicating intralesional hemorrhage was observed on T2WI (A, arrow), T1WI (D, arrow), and DWI, with *b* = 0 s/mm^2^ (F, arrow). The pathological report disclosed atypical teratoid rhabdoid tumor with focal tumor necrosis.

We further evaluated intratumoral morphology, including areas with necrosis, cysts, hemorrhage, and calcification as well as the dominant drainage veins for both tumor types. Intratumoral hemorrhage and calcification were recorded by comprehensively evaluating the T1WI, T2WI, DWI (*b* = 0 s/mm^2^), GRE, and SWI findings.^19^, ^20^, ^21^, ^22^, ^23^ Intratumoral hemorrhage was defined as signal alterations characteristic of hematoma and the presence of fluid–fluid levels (Figure 2).^19^ Most calcifications show low signals on both T1WI and T2WI, but some calcifications with large surface areas may appear hyperintense on T1WI.^20^ In addition, both hemorrhage and calcification appear hypointense on DWI (*b* = 0 s/mm^2^), GRE and SWI.^21^, ^22^, ^23^, ^24^ Suspected hemorrhage and calcification on MRI were further confirmed by CT, surgical reports and pathological findings.

**FIGURE 3:**
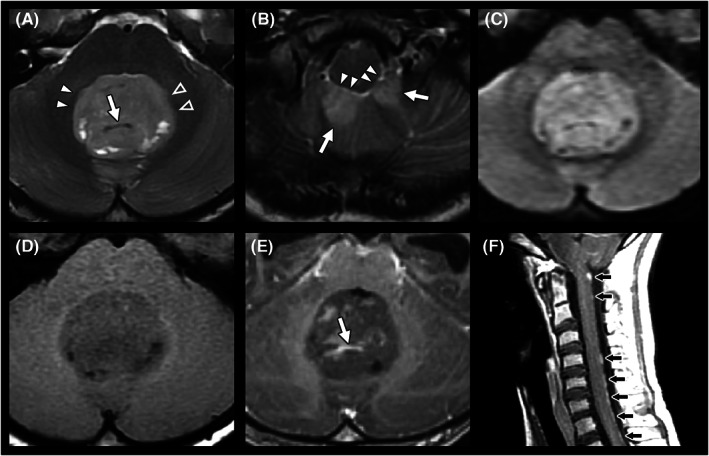
A case of medulloblastoma. This is a 10‐year boy with medulloblastoma. T2WI (A, B) demonstrated a hyperintense mass lesion from bilateral cerebellar tonsils (b, arrows) with upward extension to the fourth ventricle (A). The tumor had clear margins without invasion into the right middle cerebellar peduncle (A, arrowheads) or the brainstem (B, arrowheads). A fuzzy margin indicating local tumor invasion was noted at the left middle cerebellar peduncle (A, open arrowheads), which was later proven by documentation of a left infiltrating tumor in the surgical report. The lesion was hypointense on T1WI (D), showed heterogeneous enhancement after administration of the contrast agent (E), and was hyperintense in relation to the cerebellar white matter on DWI, *b* = 1000 s/mm^2^ (C). A dominant central drainage vein was observed on T2WI (A, arrow) and contrast‐enhanced T1WI (E, arrow). In addition, leptomeningeal seeding was noted on sagittal contrast‐enhanced T1WI (F, black arrows).

**FIGURE 4:**
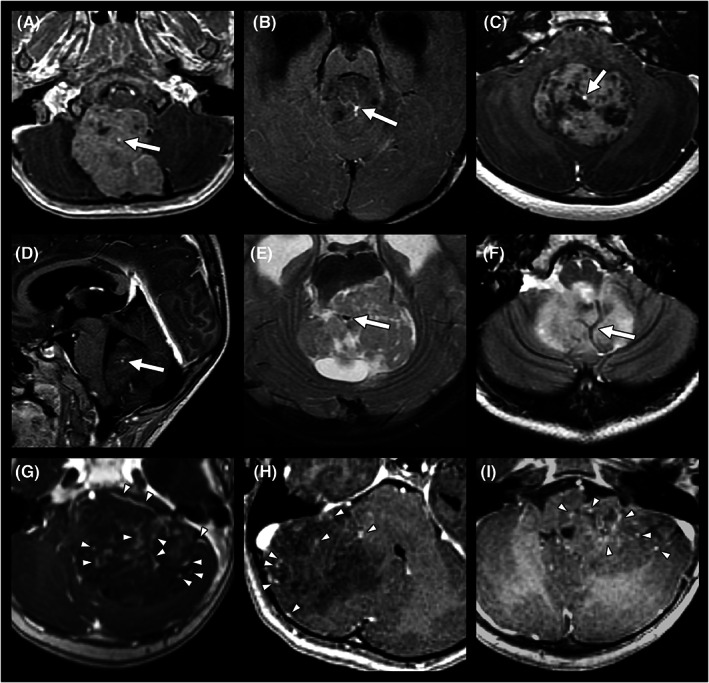
The tumor central vein sign. (A–F) represents six different patients with medulloblastomas. A single dominant central drainage vein (arrows) was observed on axial (A–C) and sagittal (D) contrast‐enhanced T1WI and axial T2WI (E, F), indicating the “tumor central vein sign”. In comparison, (G–I) shows three different patients with AT/RTs. Multiple scattered small drainage veins (arrowheads) with both peripheral and central distributions were observed on contrast‐enhanced T1WI (G–I).

The distribution of the main tumor drainage veins at either the central or peripheral location was evaluated on contrast‐enhanced T1WI and T2WI (appearing as flow voids). The “tumor central vein sign” was defined as a single, dominant central intratumoral drainage vein that was clearly visible on contrast‐enhanced T1WI and/or T2WI. The vein should be located centrally in the tumor, regardless of the lesion's shape, and may either appear as a thin line or dot. (Figures 3 and 4).

We also evaluated peritumoral involvement in pediatric patients with posterior fossa tumors. Peritumoral invasion into the adjacent brainstem and middle cerebellar peduncle was recorded by closely observing the fuzzy margins between the tumor and brain parenchyma on T2WI and contrast‐enhanced T1WI.^25^ (Figures 2 and 3) Peritumoral brain edema at the cerebellar peduncle or cerebellum was recorded. We also observed downward transforaminal extension of the tumor below the foramen magnum (below the McRae line), hydrocephalus (Evans' index >0.3) and leptomeningeal seeding (Figure 3F).^24^, ^26^ Those with intraventricular drainage tubes were excluded from the analysis of ventricular dilatation.

### 2.3 Postsurgical imaging studies

Postsurgical MRI findings were evaluated in all patients. The follow‐up tumor status (recurrence/progression, stationary, or regression) was analyzed; two medulloblastoma patients with irradiation‐induced gliomas were classified as having tumor progression. In those with tumor recurrence or progression, we evaluated the lesion patterns (local recurrence/progression, leptomeningeal seeding, or both).

### 2.4 Statistical assessment

All statistical analyses were performed with IBM® SPSS® software. Continuous variables are summarized as the mean values with standard deviations; P values were calculated with the Mann–Whitney U test. Categorical variables are summarized as counts and percentages. P values were calculated with Pearson's chi‐square or Fisher's exact test for factors with two categorical variables and with likelihood ratio tests for variables with more than 3 categories. Patient survival time was presented by using Kaplan–Meier method and P values were calculated with log‐rank test. The optimal cutoff levels of the DWI ratio and ADC ratio for differentiating AT/RT and medulloblastoma were analyzed by receiver operating characteristic (ROC) curves and Youden's index. Patients with missing data for a variable were excluded from the analysis of that specific variable. All reported P values are two‐sided. P values of less than 0.05 are regarded as statistically significant (in bold).

## 3 RESULTS

### 3.1 Demographic features

The characteristics of the 16 AT/RT patients and 57 medulloblastoma patients are provided in Table 1. The two groups did not differ in terms of sex, surgery, or adjuvant treatment of the primary tumor. AT/RTs were diagnosed at a significantly younger age than medulloblastomas (2.8 ± 4.9 [0–17] vs. 6.5 ± 4.0 [0–18], *p* < 0.001).

**TABLE 1:** Demographic features of the 73 pediatric patients with primary atypical teratoid/rhabdoid tumor or medulloblastoma of the posterior fossa.

Demographic features	Atypical teratoid rhabdoid tumor (*N* = 16)	Medulloblastoma (*N* = 57)	*p* value
Female sex—no. (%)	8 (50.0)	23 (40.4)	0.57
Age at the initial diagnosis of primary tumor—years^a^	2.8 ± 4.9 (0–17)	6.5 ± 4.0 (0–18)	**<0.001**
Surgical treatment of primary tumor			0.20
Gross total removal—no. (%)	2 (12.5)	19 (33.3)	
Nearly total removal—no. (%)	7 (43.8)	23 (40.4)	
Subtotal or partial removal—no. (%)	7 (43.8)	15 (26.3)	
Adjuvant treatment of primary tumor			
Chemotherapy—no. (%)	15 (93.8)	55 (96.5)	0.53
Radiotherapy—no. (%)	13 (81.3)	53 (93.0)	0.17
Follow‐up tumor status			**0.003**
Tumor recurrence or progression—no. (%)	13 (81.3)	20 (35.1)	
Stationary disease—no. (%)	0 (0)	2 (3.5)	
Disease regression—no. (%)	3 (18.8)	35 (61.4)	
Recurrent cases and pattern	12 (75.0%)	20 (35.1%)	0.86
Local recurrence/progression—no. (%)	4 (30.8)	8 (40.0)	
Leptomeningeal seeding—no. (%)	5 (38.5)	7 (35.0)	
Local recurrence/progression and distant seeding—no. (%)	4 (30.0)	5 (25.0)	
Interval between surgery and recurrence/progression—month^a^	8.5 ± 10.6 (1–33)	32.0 ± 40.9 (3–137)	**0.004**
Follow‐up period—month^a^	30.5 ± 47.4 (1–191)	76.8 ± 55.2 (2–187)	**<0.001**
Survival^b^			**0.005**
Alive—no. (%)	3 (21.4)	35 (66.0)	
Deceased—no. (%)	11 (78.6)	18 (34.0)	
Survival time—month^a^ ^,^ ^b^	45.5 ± 17.7 (3–191)	128.4 ± 10.8 (10–187)	**<0.001**

^a^ Data are expressed as the mean ± standard deviation (range).

^b^ Two patients with atypical teratoid rhabdoid tumors and four patients with medulloblastoma were lost to long‐term follow‐up and thus were excluded from the calculations.

### 3.2 clinical outcomes

During the follow‐up period, AT/RTs had a significantly higher incidence of disease progression than medulloblastomas (13/16 [81.3%] vs. 20/57 [35.1%], *p* = 0.003). Among those with progressive disease, the time interval from surgery to tumor recurrence/progression was significantly shorter in patients with AT/RT (8.5 ± 10.6 [1–33] months) than in patients with medulloblastoma (32.0 ± 40.9 [3–137] months) (*p* = 0.004). No significant difference was observed in the recurrence pattern (local recurrence/progression or leptomeningeal seeding) between the two groups (*p* = 0.86).

Two AT/RT and four medulloblastoma patients were lost to long‐term follow‐up. The overall survival time in the remaining patients was 45.5 ± 17.7 (3–191) months in AT/RT patients and 128.4 ± 10.8 (10–187) months in medulloblastoma patients (*p* < 0.001). By the end of the study, AT/RT was associated with a significantly higher mortality rate than medulloblastoma (78.6% [11/14] vs. 34.0% [18/53], *p* = 0.005).

### 3.3 MRI findings

**FIGURE 5:**
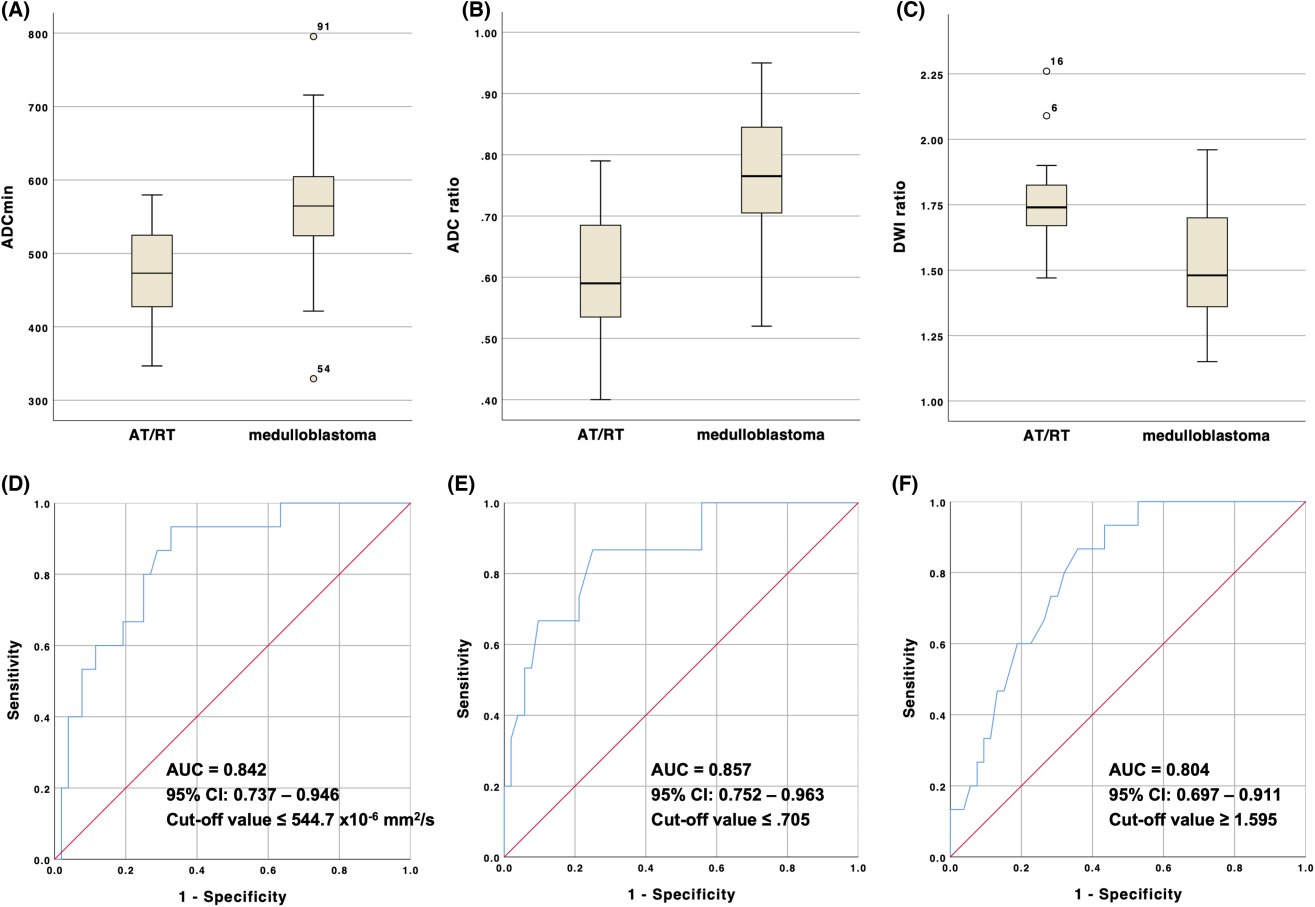
ADC_min_, ADC ratio and DWI ratio of AT/RT vs. medulloblastoma. Box plots and ROC curves demonstrated a lower ADC_min_ in AT/RT (A, D), a lower ADC ratio in AT/RT (B, E), and a higher DWI ratio in AT/RT (C, F). Outliers higher than the 75th percentile or lower than the 25th percentile in the box plots are marked as open circles. AUC, area under the curve.

MRI features of the 16 AT/RTs and the 57 medulloblastomas are provided in Table 2. In the presurgical MRI examination, no difference was observed in lesion size or signal intensity on T1WI, T2WI or contrast‐enhanced T1WI between the two groups. DWI was performed in 15 AT/RT and 53 medulloblastoma patients. The raw data for calculating the ADC were accessible for 15 AT/RT and 52 medulloblastoma patients. AT/RT was associated with a significantly lower ADC_min_ than medulloblastoma (469.4 ± 66.9 [347–580] vs. 565.0 ± 76.9 [329–795] × 10^−6^ mm^2^/s, *p* < 0.001), as well as a significantly lower ADC ratio (0.61 ± 0.11 [0.40–0.79] vs. 0.77 ± 0.10[0.52–0.95], *p* < 0.001) and a higher DWI ratio (1.77 ± 0.20 [1.47–2.26] vs. 1.52 ± 0.21 [1.15–1.96], *p* < 0.001) (Figures 1 and 5). In the ROC curve analysis, the ADC_min_ cutoff value for differentiating AT/RT from medulloblastoma was 544.7 × 10^−6^ mm^2^/s, with values under the cutoff indicative of AT/RT; the cutoff had 93.3% sensitivity and 67.3% specificity, and the area under the curve (AUC) was 0.842 (Figure 5A,D). The ADC ratio had a cutoff value of 0.705 for differentiating AT/RT from medulloblastoma, with values under the cutoff indicative of AT/RT; the cutoff had 86.7% sensitivity and 75.0% specificity, and the AUC was 0.857 (Figure 5B,E). The DWI ratio had a cutoff value of 1.595 for differentiating AT/RT from medulloblastoma, with values under the cutoff indicative of AT/RT; the cutoff had 86.7% sensitivity and 64.2% specificity, and the AUC was 0.804 (Figure 5C,F). No difference was seen in the ADC and DWI values of normal white matter between the groups.

**TABLE 2:** MRI features of the 73 pediatric patients with primary atypical teratoid rhabdoid tumor or medulloblastoma.

Image features	Atypical teratoid rhabdoid tumor (*N* = 16)	Medulloblastoma (*N* = 57)	*p* value
Tumor size—mm^a^	48.3 ± 12.2 (28–72)	46.6 ± 10.2 (20–77)	0.053
Tumor signals			
T1WI			0.14
Hyperintensity or isointensity—no. (%)	5 (31.3)	8 (14.0)	
Hypointensity—no. (%)	11 (68.8)	49 (86.0)	
T2WI			0.57
Hyperintensity—no. (%)	8 (50.0)	34 (59.6)	
Isointensity or hypointensity—no. (%)	8 (50.0)	23 (40.4)	
Contrast‐enhanced T1WI^b^			0.23
Well‐enhancement—no. (%)	14 (87.5)	50 (96.2)	
No/poor enhancement—no. (%)	2 (12.5)	2 (3.8)	
ADC_min_—10^−6^ mm^2^/s^a^ ^,^ ^c^	469.4 ± 66.9 (347–580)	565.0 ± 76.9 (329–795)	**<0.001**
ADC—Tumor/contralateral white matter ratio^a^ ^,^ ^c^	0.61 ± 0.11 (0.40–0.79)	0.77 ± 0.10 (0.52–0.95)	**<0.001**
DWI—Tumor/contralateral white matter ratio^a^ ^,^ ^c^	1.77 ± 0.20 (1.47–2.26)	1.52 ± 0.21 (1.15–1.96)	**<0.001**
Intratumoral morphology			
Tumor Central vein sign—no. (%)	1 (6.3)	24 (42.1)	**0.007**
Necrosis—no. (%)	13 (81.3)	33 (57.9)	0.14
Cysts—no. (%)	15 (93.8)	51 (89.5)	1.00
Hemorrhage—no. (%)	6 (37.5)	8 (14.0)	0.066
Calcification—no. (%)	7 (43.8)	11 (19.3)	0.056
Peritumoral and distant involvement			
Peritumoral brain invasion			
Brainstem—no. (%)	12 (75.0)	10 (17.5)	**<0.001**
Middle cerebellar peduncle—no. (%)	15 (93.8)	16 (28.1)	**<0.001**
Peritumoral brain edema—no. (%)	4 (25.0)	28 (49.1)	0.10
Downward transforaminal extension—no. (%)^d^	4 (25.0)	17 (29.8)	1.00
Leptomeningeal seeding—no. (%)	5 (31.3)	11 (19.3)	0.32
Hydrocephalus—no. (%)^e^	6 (42.9)	39 (69.6)	0.12

Abbreviations: ADC, apparent diffusion coefficient; DWI, diffusion‐weighted imaging; T1WI, T1‐weighted imaging; T2WI, T2‐weighted imaging.

^a^ Data are expressed as the mean ± standard deviation (range).

^b^ Contrast‐enhanced T1WI was not performed in five medulloblastoma patients; thus, they were excluded from the calculation.

^c^ DWI was not performed in 1 AT/RT and 4 medulloblastoma patients, and ADC was not performed in 1 AT/RT and 5 medulloblastoma patients; thus, they were excluded from the calculation.

^d^ Defined by transforaminal extension of the tumors below the foramen magnum (below the McRae line).

^e^ Defined by Evans' index >0.3. Patients with ventricular drainage tubes were excluded from the calculation.

Regarding the intratumoral morphology, the “tumor central vein sign” was significantly more frequently observed in medulloblastomas than in AT/RTs (24/57 [42.1%] vs. 1/16 [6.3%], *p* = 0.007) (Figures 3 and 4). AT/RTs had higher incidence rates of intratumoral hemorrhage (6/16 [37.5%] in AT/RT vs. 8/57 [14.0%] in medulloblastoma, *p* = 0.066) (Figure 2) and intralesional calcification (7/16 [43.8%] in AT/RT vs. 11/57 [19.3%] in medulloblastoma, *p* = 0.056). According to the analysis of peritumoral invasion on T2WI, AT/RTs were significantly more prone to invade the brainstem (12/16 [75.0%] vs. 10/57 [17.5%], *p* < 0.001) and middle cerebellar peduncle (15/16 [93.8%] vs. 16/57 [28.1%], *p* < 0.001) (Figure 2).

## 4 DISCUSSION

Although AT/RT and medulloblastoma of the posterior fossa have been reported to have similar clinical features and are nearly indistinguishable on neuroimaging, this study presents distinct clinical and MRI features that differentiate these two pediatric disease entities.^5^, ^11^, ^12^, ^13^, ^27^ In comparison to medulloblastoma, AT/RT has a higher progression/recurrence rate as well as a higher mortality rate. On MRI, AT/RT has a significantly lower ADC_min_ and ADC ratio and a higher DWI ratio and is more prone to peritumoral invasion on T2WI. On the other hand, the “tumor central vein sign” had a significantly higher incidence in medulloblastoma than in AT/RT.

DWI and the ADC are basic factors that are routinely obtained in MRI of brain tumors. By measuring the random movement of intralesional water molecules, their signal characteristics can reflect the cellularity and grading of various tumors.^12^, ^17^ Reported ADC values in young patients with AT/RT or medulloblastoma from an English literature review are listed in Table 3.^11^, ^12^, ^13^, ^14^, ^18^, ^28^, ^29^, ^30^, ^31^ Although the difference between embryonal tumors and other tumor types was disclosed, none of the studies were able to distinguish AT/RT from medulloblastoma using the ADC value, which may be related to the small number of cases. Gauvain et al,^28^ Rumboldt et al,^13^ Ahmeda et al^18^ and Phuttharak et al^12^ reported a lower ADC_min_ or ADC ratio in AT/RT cases than medulloblastoma cases, which is consistent with our study. Notably, we are the first to demonstrate the differences statistically. The ADC_min_ values reported by Koral et al^14^ and Yamashita et al^29^ were higher in AT/RT cases than in medulloblastoma cases, although only six and one AT/RT patients were included in their studies, respectively.

**TABLE 3:** English literature review of the ADC_min_ values and ADC ratios of cranial AT/RTs and medulloblastomas in young patients.

Study	Year	Patient number	Patient age (yrs)	ADC_min_ (x10^−3^ mm^2^/s)^a^	ADC ratio^a^	Size of tumor ROI	Normal reference for calculating ADC ratio
Atypical teratoid rhabdoid tumor (AT/RT)							
Gauvain et al^28^, ^b^	2001	1	2	0.60	0.70	N/A	Contralateral homologous brain regions
Rumboldt et al^13^	2006	2	1 and 1	0.56 and 0.63	0.64 and 0.74	50–100 mm^2^	Cerebellar white matter and centrum semiovale
Koral et al^14^	2008	6	≤1	0.55 ± 0.06 (0.45–0.60)	N/A	15 pixels (diameter)	N/A
Yamashita et al^29^	2013	1	23	0.57	N/A	~ 30 mm^2^	N/A
Jin et al^11^, ^c^	2013	9	0–9	0.60 ± 0.13 (0.46–0.85)	N/A	30–70 mm^2^	Contralateral white matter
Ahmeda et al^18^	2018	2	≤15	0.69 ± 0.00 (N/A)	0.87 ± 0.01 (N/A)	N/A	Cerebellum or brain stem
Phuttharak et al^12^, ^d^	2021	4	≤19	N/A.	0.86 ± 0.16 (0.63–1)	N/A	Cerebellar white matter
Present study		15	0–17	0.47 ± 0.07 (0.35–0.58)	0.61 ± 0.11 (0.40–0.79)	10–50 mm^2^	Contralateral cerebellar white matter
Medulloblastoma							
Gauvain et al^28^	2001	2	13 and 14	0.54 and 0.78	0.92 and 1.22	N/A	Contralateral homologous brain regions
Rumboldt et al^13^	2006	8	0–23	0.66 ± 0.15 (0.48–0.93)	0.84 ± 0.14 (0.66–1.10)	50–100 mm^2^	Cerebellar white matter and centrum semiovale
Koral et al^14^	2008	14	≤14	0.47 ± 0.16 (0.27–0.83)	N/A	15 pixels (diameter)	N/A
Yamashita et al^29^	2013	11	0–25	0.49 ± 0.06 (0.39–0.54)	N/A	~ 30 mm^2^	N/A
Pierce et al^30^	2014	33	6.4 ± 4.6^a^	0.54 ± 0.09 (N/A)	0.70 ± 0.12 (N/A)	30 mm^2^	Putamen
Zitouni et al^31^	2017	18	7.38 ± 4.14^a^	0.71 ± 0.21 (0.51–1.25)	1.02 ± 0.30 (N/A)	~100 mm^2^	Cerebellar parenchyma
Ahmeda et al^18^	2018	9	≤15	0.70 ± 0.12 (N/A)	0.96 ± 0.21 (N/A)	N/A	Cerebellum or brain stem
Phuttharak et al^12^, ^d^	2021	24	1–19	N/A	0.91 ± 0.17 (0.64–1.45)	N/A	Cerebellar white matter
Present study		53	0–18	0.57 ± 0.08 (0.33–0.80)	0.77 ± 0.10 (0.52–0.95)	10–50 mm^2^	Contralateral cerebellar white matter

^a^ Data are expressed as the mean ± standard deviation (range).

^b^ This lesion was supratentorial.

^c^ Five tumors among them were supratentorial.

^d^ Phuttarak et al used a “5‐point DWI visual scale” instead of the DWI ratio in measuring the tumor signal intensity on DWI. All (4/4) AT/RTs were markedly hyperintense, while 25% (6/24) of medulloblastomas were hyperintense, and 75% (18/24) of medulloblastomas were markedly hyperintense.

All AT/RT cases reported in the literature had an ADC_min_ ranging from 0.45 to 0.85 (×10^−3^ mm^2^/s) and an ADC ratio ranging from 0.63 to 0.87; our study revealed an ADC_min_ of 0.47 ± 0.07 (0.35–0.58) and an ADC ratio of. 0.61 ± 0.11 (0.40–0.79) in AT/RT cases. The reported medulloblastoma cases had an ADC_min_ ranging from 0.27 to 1.25 (×10^−3^ mm^2^/s) and an ADC ratio ranging from 0.64 to 1.45; in comparison, our study revealed an ADC_min_ of 0.57 ± 0.08 (0.33–0.80) (×10^−3^ mm^2^/s) and ADC ratio of 0.77 ± 0.10 (0.52–0.95) in medulloblastoma cases. The ADC values measured in our study are quite similar but slightly lower than those in the literature. Notably, as we placed the tumor ROI at the most homogenous hypointense solid part of the lesion (Figure 1), the area of the ROI may be as small as 10 mm^2^. The benefit of our small ROI in measuring the most homogeneous hypointense part of the tumor includes its easy visualization and its representation of the most malignant tumor focus. Analyzing the most malignant tumor focus allows more accurate clinical tumor grading and diagnosis. In comparison, the areas of tumor ROIs in the literature were larger than that in our study and ranged from 30 to 100 mm^2^ (Table 3). Large area measurements may include more uneven cell structures that result in a higher mean ADC_min_ value. Furthermore, the normal reference used for calculating the ADC ratio differed between studies (Table 3). The majority of the studies used cerebellar white matter as the reference, similar to our study. Additionally, many of our referential ROIs were positioned at the middle cerebellar peduncle (Figure 1). In contrast, some studies chose “cerebellar parenchyma” or “homogenous brain regions” as normal references instead of precisely placing the ROI at the white matter, which may correspondingly lead to a higher ADC ratio.

The DWI ratio has been previously used by Wu et al^17^ in tumor grading of brain gliomas, but it has never been applied in embryonal brain tumors. Phuttharak et al^12^ created a “five‐point visual scale” for analysis of the DWI intensity of posterior fossa tumors. All (4/4) AT/RTs were markedly hyperintense, while 25% (6/24) of medulloblastomas were hyperintense and 75% (18/24) of medulloblastomas were markedly hyperintense. This is consistent with our finding that AT/RTs were more hyperintense on DWI than medulloblastomas (DWI ratio: 1.77 ± 0.20 [1.47–2.26] vs. 1.52 ± 0.21 [1.15–1.96], *p* < 0.001). Both the DWI and ADC data of the present study support the notion that the tumor cell grade of AT/RT is higher than that of medulloblastoma.

A recent report by Zhang et al^27^ successfully distinguished AT/RT from medulloblastoma via machine learning by analyzing MRI‐based radiomic phenotypes, including their morphology and signal intensities on T2WI and contrast‐enhanced T1WI. Their finding is key breakthrough, but these radiometric features are indiscernible to the human eye, and the high technical requirement limits routine clinical application. Therefore, we propose the following differentiating characteristics: the “tumor central vein sign” for medulloblastoma, a lower ADC value/ratio for AT/RT, and more aggressive marginal invasion for AT/RT. Our findings can be easily applied and interpreted in daily clinical practice and can assist future radiomics research.

Additionally, we found a higher incidence of intralesional hemorrhage in AT/RTs than in medulloblastomas, which is in accordance with a previous report by Koral et al.^14^ In addition, AT/RTs were reported to have a higher cerebral blood volume (CBV) value than medulloblastomas on perfusion MRI by Goo et al,^32^ indicating more abundant neovascularity in AT/RTs, which may support our finding. Abundant tumor vascularity may be unfavorable for complete surgical resection, thus influencing the outcomes of AT/RT patients, especially those who are very young.

A dominant central drainage vein was observed in 42.1% of the medulloblastomas but in only 1 AT/RT (6.3%). Although large draining veins in medulloblastomas were observed by neurosurgeons during tumor resection in one report,^33^ the “tumor central vein sign” on MRI has never been highlighted in the literature. The presence of this dominant drainage vein in medulloblastomas may allow an accurate pretreatment diagnosis and avoid potential massive intraoperative tumor hemorrhage.

In our study, AT/RT was associated with younger age, more aggressive clinical behavior and a worse outcome than medulloblastoma, these were compatible with the findings in previously reported literatures.^9^, ^10^ The dismal prognosis of AT/RT can be explained by several aspects observed in the present study: (1) The majority of AT/RTs occurred in very young children. Their unfused skull sutures accommodate the progressively increasing cranial pressure during tumor growth, which results in delayed symptom onset.^34^ Even when symptoms are present, some nonspecific manifestations (irritability, vomiting, etc.) can be difficult to express due to the age of these very young patients, which results in diagnostic delay^34^, ^35^; (2) Surgery, chemotherapy and radiotherapy are regarded as the standard treatments for AT/RT. Maximal surgical resection and aggressive adjuvant therapy are particularly crucial in AT/RT treatment due to its invasive behavior.^27^ However, aggressive surgery followed by irradiation is sometimes postponed in patients under 3 years of age due to neurological side effects, which may lead to suboptimal treatment and a poor outcome.^36^, ^37^ (3) Significantly greater peritumoral invasion into the brainstem and cerebellum by AT/RT than by medulloblastoma was observed on T2WI, and AT/RT can leave subclinical residual tumors after surgical resection. Without aggressive adjuvant treatment, these subclinical residual tumors at the tumor bed or brain surface may become the origin of tumor recurrence or leptomeningeal seeding. In addition, more tumor bleeding in AT/RTs, as noted in the present study, and the reported higher tumor vascularity of AT/RTs may increase the technical difficulty of complete resection. (4) In the present study, on MRI, AT/RTs had significantly higher DWI ratios than medulloblastomas. A higher DWI value in CNS gliomas was proven to be associated with a higher tumor grade and a worse prognosis.^17^, ^38^ These aggressive tumor behaviors may explain the significantly lower survival rate in AT/RT patients than in medulloblastoma patients.

There are some limitations to our study. First, this was a retrospective study, with a relatively small number of AT/RT patients due to the rarity of the disease. Second, while statistically significant, the sensitivity and specificity of some radiologic findings may not reach high clinical significance. Surgical intervention and pathological diagnosis are still important for patients with AT/RT and medulloblastoma. Third, molecular analysis was not performed in our study. Of note, WNT/SHH subgrouping was not widely used until the pronouncement of the 2016 WHO classification of CNS tumors.^39^ Further study is recommended. Forth, the MR images were obtained from different scanners, which may influence ADC and DWI measurements. Therefore, we further calculated the ADC ratios and DWI ratios to offset the different settings between scanners. Fifth, we did not evaluate more advanced MRI techniques, such as perfusion imaging and MR spectroscopy.^32^

## 5 CONCLUSION

AT/RT occurs at a younger age, has more aggressive clinical behavior, and has a more indistinct tumor margin than medulloblastoma, which results in a much worse prognosis. Maximal surgical resection and aggressive adjuvant therapy are therefore important in AT/RT treatment. We can differentiate AT/RT from medulloblastoma on MRI by the lower ADC value, higher degree of peritumoral invasion, and absence of the “tumor central vein sign,” These MRI characteristics may be helpful in making pretreatment diagnosis and appropriate outcome evaluation.

## AUTHOR CONTRIBUTIONS

**Hsin‐Wei Wu:** Data curation (equal); formal analysis (lead); investigation (lead); methodology (equal); validation (equal); visualization (lead); writing – original draft (lead). **Chia‐Hung Wu:** Funding acquisition (lead); methodology (supporting); project administration (supporting); supervision (equal); writing – review and editing (supporting). **Shih‐Chieh Lin:** Investigation (equal); resources (equal); supervision (supporting); writing – original draft (supporting); writing – review and editing (supporting). **Chih‐Chun Wu:** Supervision (supporting); writing – review and editing (supporting). **Hsin‐Hung Chen:** Supervision (supporting); writing – review and editing (supporting). **Yi‐Wei Chen:** Supervision (supporting); writing – review and editing (supporting). **Yi‐Yen Lee:** Supervision (supporting); writing – review and editing (supporting). **Feng‐Chi Chang:** Conceptualization (lead); data curation (equal); formal analysis (equal); funding acquisition (lead); investigation (supporting); methodology (lead); project administration (lead); resources (equal); supervision (lead); validation (equal); visualization (equal); writing – original draft (equal); writing – review and editing (lead).

## CONFLICT OF INTEREST STATEMENT

The authors declare that they have no competing interests.

## ETHICAL APPROVAL STATEMENT

Informed consent to perform imaging examinations, surgery and adjuvant cancer treatment was obtained from each patient or their family. This retrospective study was approved and deemed exempt from individual patient consent for this research project by the institutional review board of our institute.

## CLINICAL TRIAL REGISTRATION NUMBER

This retrospective study was approved by the institutional review board of Taipei Veterans General Hospital (IRB‐TPEVGH No.: 2022–07‐020 BC).

## Data Availability

The datasets generated during and/or analyzed during the current study are available from the corresponding author on reasonable request.
